# Chromosome 20p Partial *De Novo* Duplication Identified in a Female Paediatric Patient with Characteristic Facial Dysmorphism and Behavioural Anomalies

**DOI:** 10.1155/2020/7093409

**Published:** 2020-07-11

**Authors:** Shahzaib Khattak, Meryam Jan, Sara Warsi, Sohail Khattak

**Affiliations:** ^1^The Kids Clinic, 300 Rossland Rd E, Suite 301, Ajax, ON L1Z 0M1, Canada; ^2^McMaster University, 1280 Main St W, Hamilton, ON L8S 4L8, Canada

## Abstract

Copy number variations (CNVs) involving the *JAG1* gene are rare and infrequently reported in the scientific literature. Recently, a generally healthy young patient presenting with a history of behavioural concerns was referred to us. Herein, we discuss the patient, a 7-year-old female possessing a 0.797 Mb microduplication within the short arm of chromosome 20 at band 12.2. The patient generates considerable curiosity due to the rarity of her case, which includes a *de novo* partial duplication involving the *JAG1* gene. The patient exhibits a wide range of symptoms including facial dysmorphism (dolichocephaly, round face, tented philtrum, anteverted nares, and micrognathia), clinodactyly, and an inborn congenital heart defect. She presented with behavioural concerns including ADHD-I, SPD, motor clumsiness, and poor self-regulation. Deletions in *JAG1* are often linked to *Alagille Syndrome*; however, complete duplications have not been specifically identified as disease-causing. *JAG1* mutations are reported alongside various clinical features including facial dysmorphology, heart defects, vertebral abnormalities, and ocular dysmorphic features (strabismus, epicanthal folds, and slanted palpebral fissures). This particular microduplication is rare, and thus, limited data exist regarding its significance. To our knowledge, most reported duplications are larger than 0.797 Mb. This may define a critical region causing phenotypical changes in some patient cases.

## 1. Introduction

Information concerning *de novo* genetic aberrations on the short arm of chromosome 20 is absent from the current scientific literature. *JAG1* mutations are primarily associated with Alagille syndrome [1–5] caused by microdeletions. Also, variations in JAG1 are associated with multiple types of cancer including breast cancer and adrenocortical carcinoma [6, 7]. Pure trisomy 20p is infrequently reported [8, 9]. Clinical characteristics of mutated 20p with regard to *JAG1* are seen in Alagille syndrome [6] and are correlated with deregulation of *NOTCH* signaling. *JAG1* acts as a ligand in the NOTCH signaling pathway, which plays an important role in cell signaling and cell membrane protein transportation. NOTCH is a key component of embryogenesis, organogenesis, and cell fate specification [10]. Since *JAG1* acts as a ligand in the NOTCH signaling pathway, *JAG1* gene deletions would downregulate the Notch signaling [11]. *JAG1* deletions risk interrupting the NOTCH signaling pathway. 20p duplications are less common than deletions and typically show a wider range of phenotypic traits. Variability is based on the size of the duplicated segment. Symptoms include, but are not limited to, developmental delay, round facial structure, prominent cheeks, and ocular abnormalities (slanted palpebral fissures or strabismus) [8, 9, 11, 12].

Our focus of discussion is a rare *de novo* 20p microduplication at band 12.2 seen in a young female patient with delayed speech and intellectual disability (ID) (based on DSM-5 criteria, our patient suffers from intellectual disability (intellectual development disorder) severity level: mild DSM-5 Code F70). ID is a disorder involving intellectual and adaptive functioning deficits with onset during the developmental period. Intellectual functions involve deficiencies in reasoning, problem solving, abstract thinking, etc. with IQ scores ranging between 65 and 70. Adaptive functions involve failing to adhere to developmental and social cultural standards, poor motor skills, ADHD (*predominantly inattentive presentation*) (ADHD-I), sensory processing disorder, characteristic dysmorphia, and congenital heart defects.

## 2. Case Presentation

In February 2016, the patient was referred to our clinic for a routine evaluation of behavioural issues. Her age, at this time, was 7 years and 3 months. The main concerns regarding the patient were behavioural issues such as an inability to focus, frequent temper tantrums, sensitivity to loud noises, and foul language. She was a generally healthy child with no seizures, medications, or recent hospitalizations. The patient was conceived via intrauterine insemination (IUI) as a twin pregnancy between nonconsanguineous Caucasian parents. The mother underwent amniocentesis during pregnancy. Developmental history showed prenatal complications involving a blood clot at 7 months of gestation. For the remainder of the pregnancy, the mother was put on Fragmin (dalteparin). Fetal karyotypes were reportedly normal. At 37 weeks of gestation, the mother was induced and she had a vaginal delivery. Further complications arose during delivery when it was noticed that the arm was positioned above the head, requiring vacuum extraction. In the postpartum period, the patient's haematology panel showed hypoglycaemia. She was kept in a neonatal intensive care unit (NICU) for 36 hours after delivery. The blood sugar recovered in the first 12 hours. The patient was observed for an additional 24 hours and was discharged thereafter. Her birth weight was recorded at 5 pounds, 10 ounces. Apgar scores were normal. At birth, an abdominal and renal ultrasound showed stasis nephropathy. Subsequently, a voiding cystourethrogram (VCUG) was done and it showed normal renal structure with no reflux.

**Figure 1:**
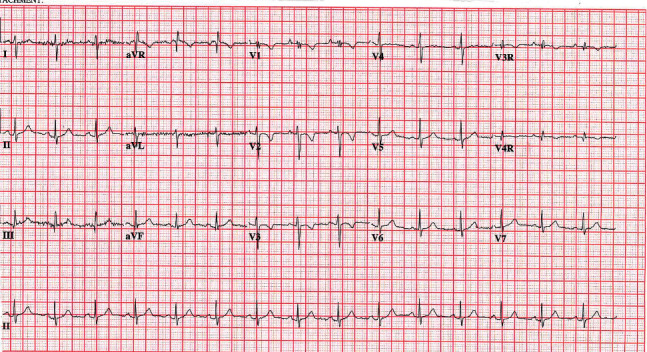
Patient's abnormal EKG ventricular conduction delay (crochetage).

**Figure 2:**
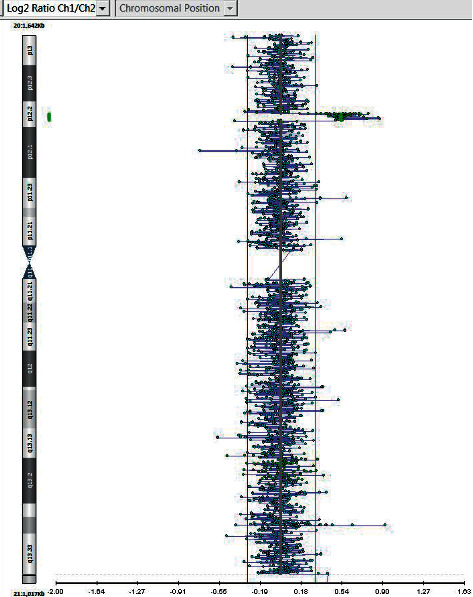
Detail of the array CGH showing duplication of chromosome 20 (0.797 Mb) in the patient. Chromosomal view showing a deviation of baseline (log 2 ratio of −2). Image obtained from Credit Valley Hospital.

The patient was noted to have facial dysmorphic features and a heart murmur. She went through extensive investigation and was diagnosed with atrial septal defect (ASD) (secundum), which was subsequently repaired due to a substantial left to right shunt and right ventricular (RV) dilation at the age of 4. She had a persistent minor ventricular conduction delay (crochetage) in the right precordial leads due to ASD (Figure 1). The patient was noted to have facial dysmorphic features including anteverted nares, short nose, tented philtrum, round face, micrognathia, epicanthic folds, unilateral ptosis, and clinodactyly (Table 1). Strabismus and unilateral congenital ptosis were surgically corrected. Additionally, the patient had myringotomy tube insertions twice. Her head circumference in 2016 at age 8 was measured to be 53.1 cm (90^th^ percentile). Her height was measured to be 115.1 cm (5^th^ percentile), and her weight was 23.3 kg (50^th^ percentile). The patient's facial dysmorphic features and ASD resulted in clinical genetic consultation in 2011. Chromosomal karyotyping of peripheral blood was performed using standard protocols. Fluorescence *in situ* hybridization (FISH) was engineered using a BAC clone (RP11-103J8) which maps to chromosome 20, nucleotide position 10,840,138–10,994,545. Parental karyotypes were normal. The proband karyotype displayed gain at the 20p site, specifically registered as 46, XX, dup (20) (p12.2). The *de novo* occurrence of the microduplication was confirmed by FISH analysis that showed normal parental karyotypes. In order to determine the presence of any complex rearrangement, a genomic microarray was carried out to measure the expression levels of genomic sequences and CNVs using the genomic microarray platform CytoScan HD SNP Array (Affymetrix), genome build: NCBI 37/hg 19 (2009), and analysis software: ChAS (Affymetrix). Genomic microarray analysis showed a 0.797 Mb duplication in chromosome region 20p12.2 that involves 6 RefSeq (Reference Sequence) genes: *SLX4IP*, *MIR6870*, *LOC101929395*, *LOC101929413*, *LOC339593*, and one OMIM Morbid Map gene: *JAG1* (UCSC Genome Browser hg19). The distal breakpoint lies within the *SLX4IP* gene, resulting in partial gene duplication (Figure 2). The clinical significance of the *JAG1* duplication was indicated as unknown in the report because the literature regarding this group of CNVs was considered insufficient.

Neuroimaging was performed due to presentation of developmental delay. MRI was performed using multiplanar, multisequence imaging of the brain with axial T1, MP, RAGE and sagittal reformats, axial T2, axial proton density, diffusion imaging, coronal FLAIR, and axial T1 postgadolinium images in 2011. Results indicated dolichocephaly, with mild general volume loss. No focal intracranial abnormalities were detected.

Spinal MRI examinations were performed due to presentation of developmental delay. The technique used was sagittal T2 proton density, FIR, and T1 Angiary images of the cervical and thoracic spine with axial T2 images. Results indicated that the spine was visualized to the level of the L1 vertebral body. The cerebellar tonsils were in appropriate position. The cord was normal with normal cord signal and no cord compression. The tip of the conus was not seen, but it appeared to end just below L1. No segmentation or fusion anomalies were identified. Vertebral body heights were maintained throughout with a normal marrow signal.

The patient experienced developmental delays, where milestones were not met appropriately. She sat without support at 9 months and did not begin walking until 24 months. The patient's fine and gross motor skills, expressive and receptive language, and social skills were also delayed. The patient had difficulty forming sentences and effectively communicating with others. She struggled with social skills such as cooperatively engaging with other children and preferred parallel play. She experienced frequent mood swings and had an inability to appropriately express her feelings. Examination of the patient's family history revealed maternal anxiety and a paternal learning disability as well as a thyroid disorder. The patient's maternal grandfather and paternal grandparents were diagnosed with diabetes mellitus. Both paternal grandparents have high blood pressure and heart disease. Based on the patient's extensive family history, assessment of her liver enzymes, triglycerides, sugars, etc. was recommended. The patient's fraternal twin brother had esotropia but otherwise is healthy.

The patient's most recent assessment at 7 years and 8 months of age indicates a continuation of social skills difficulties, poor emotional regulation, and difficulty maintaining friendships/relationships with peers and the sibling. She experiences unwarranted levels of worry and anxiety and sensory sensitivity to loud noises. The patient is currently diagnosed with sensory processing disorder (SPD), mild intellectual disorder (MID), and attention deficit hyperactivity disorder. Improvements in speech and language were observed after completion of speech therapy. Her most recently recorded cardiopulmonary assessments and haematology panels are all normal.

To the best of our knowledge, the patient is the first female patient reported to have a *de novo* microduplication within the *JAG1* gene in conjunction with SPD, ID, and ADHD-I. Information on the clinical significance of the patient's chromosomal anomaly is currently unknown as there is a paucity of the published literature on *JAG1* mutations.

## 3. Discussion

We present the case of a patient, a 7-year-old female, with a rare case involving a *de novo* partial duplication in the 20p12.2 region containing the *JAG1* gene, confirmed by genomic hybridization and FISH. She did not have any other chromosomal abnormalities. The patient presented with intellectual disability, developmental delay, attention deficit hyperactivity disorder (predominantly inattentive presentation), and a sensory processing disorder. Review of medical records indicates that she had a repair of an atrial septal defect at 4 years of age. The patient exhibited classic clinical dysmorphic features of *de novo* partial 20p trisomy such as strabismus (surgically corrected), esotropia (surgically corrected), tented philtrum, and ptosis.

A microarray showed duplication to be within the short arm of chromosome 20 (10,524,474–11,322,239), as confirmed by FISH. *JAG1* is located in this region encoding a ligand in the *NOTCH1* signaling pathway. Expression of the ligand encoded by the *JAG1* gene plays a role in embryogenesis of the cardiovascular and renal systems [6].

Although microdeletion of *JAG1* is known to be syndromic, duplication of *JAG1*—as seen in our patient—has not yet been identified as a risk factor for any particular disease. Pathogenic duplications of this region have been previously reported in the ISCA and Decipher databases; however, phenotypic information is limited, and previous cases had larger duplications than those detected in our patient (0.797 Mb).

A review of the ISCA database for the patient's duplicated region of chromosome 20 displayed 3 reported losses and 11 gains with multiple size variants and phenotypes. Of the 11 gains reported, the variant size ranged from 0.49 Mb to 63 Mb. The phenotypes observed for gains in this region include developmental delay and heart abnormalities which were evident in the patient. Additional phenotypes reported in this region are microcephaly, laryngomalacia, hypotonia, aganglionic megacolon, and hydronephrosis.

Microdeletions of *JAG1* have been reported to contribute to features of Alagille Syndrome [1–5], which is a congenital disorder that clinically manifests in early childhood or infancy. One of the prominent features of the syndrome is liver damage resulting from a lack of interlobular biliary ducts [5]. Pulmonic stenosis, ventricular septal defect (VSD), ASD, and tetralogy of Fallot (TOF) are also common cardiovascular abnormalities of patients with Alagille Syndrome [5]. Missense mutations in *JAG1* resulting in VSD, ASD, and TOF have also been reported in patients that did not have Alagille Syndrome [14, 15]. *In situ* hybridization studies have previously demonstrated the importance of *JAG1* expression in the vascular structures of the heart [16], with abnormalities caused by either a dysfunction of the *NOTCH* pathway ligand *JAG1*, or potentially confounding genetic modifiers beyond the detectable *JAG1* mutation. Our patient presented with a partial duplication in *JAG1* and had an ASD that was previously repaired.

There have been very few reported cases of patients with neuropsychiatric disorders and duplications within the 20p chromosome [17]. In 2004, a case series on genomic imbalances and intellectual disabilities included a patient who presented with a *de novo* duplication within 20p12.2 containing the *JAG1* gene and suffered from mental retardation, behavioural aggression, and schizophrenia. The patient died at the age of 60 due to multiple myeloma after being institutionalized for over 40 years [18]. Our patient also displayed poor self-regulation and mild intellectual disability; however, given her age, it is uncertain whether comorbid diseases will manifest in adulthood. Chaabouni et al. [13] described a 5-year-old patient with *de novo* trisomy 20p of paternal origin of nearly the entire short arm of chromosome 20, with similar craniofacial dysmorphologies and intellectual disability as observed in the patient, including a round face with prominent cheeks and slanted palpebral fissures. Their patient also presented with hexadactyly, brachydactyly, and camptodactyly similar to our patient who presented with clinodactyly. Chaabouni's patient and our patient both had normal Apgar scores, developmental delay, moderate motor activity, and speech delay [13]. Other patient reports involving 20p duplications consistently report some of the characteristics seen in our patient (Table 1) including round face with prominent cheeks, speech delay, moderate intellectual disability, slanted palpebral fissures, and strabismus [8, 9, 12].

Upon comparing the patient to other cases we found in the literature, we observed some phenotypic features that were common to all cases and some features shared by only some (Table 1). All reported cases, including that of our patient, had the following phenotypic traits in common: round face, nasal abnormalities, neurodevelopmental delay, intellectual disability, and other behavioural issues (Table 1). In addition, most patients in the aforementioned cases shared specific facial and ocular dysmorphic features (epicanthic folds, upslanting palpebral fissures, abnormal ears, philtrum abnormalities, micrognathia, strabismus, and anteverted nares) (Table 1). Our patient and the other patients described in Table 1, displayed evidence of skull abnormalities and short necks. Finally, most patient cases involved cardiopulmonary anomalies, which in our patient's case involved a surgically repaired large ASD (Table 1). This finding is especially interesting, as other researchers have identified similar mutations in nearby genomic regions that have generated other significant cardiopulmonary syndromes. In 2013, a case report described a female patient with an approximate 770 Kb duplication in the 20p12.3 region of the genome [19]. This patient also had Wolff–Parkinson–White syndrome, which is a congenital heart disorder that can result in episodic tachycardia [19]. It is important to note that this duplication is very similar in size and in close proximity to the 0.797 Mb duplication found in the 20p12.2 region of our patient's genome. Another case report in 2013 discussed a patient with a 7.8 Mb duplication in the 20p12.2–11.23 region of the genome [20]. This patient also presented with congenital heart disease (ASD), which was also documented in our patient [20]. Comparing our patient to the cases we have explored in the literature, we hypothesize that the 20p12.2 portion of the genome may serve as a critical region that when duplicated, results in the cardiac anomalies and other defects seen in our patient and other cases of chromosome 20p duplication discussed in this report.

The aim of this case report is to contribute to the current understanding of patients with duplications within the short arm of chromosome 20, as phenotypic information on these patients is not currently well reported. Our patient presented with a *de novo* partial duplication within the DNA region containing the *JAG1* gene. As per the current literature, reported cases share common clinical features with few differences. We include an extensive genetic workup and clinical and behavioural assessments in order to assist clinicians when presented with these patients in their practices, to improve the care and management of children presenting with such genetic syndromes.

## Figures and Tables

**Table 1:** Comparison of the patient's phenotypic characteristics versus published accounts of dup 20p/trisomy 20p.

Phenotypic characteristics	Our patient	Grammatico et al. [8]^*∗*^	Bartolini et al. [9]	Trachoo et al. [12]	Chaabouni et al. [13]
Right congenital ptosis	+	−	+	−	−
Epicanthic folds	+	−	+	+	+
Upslanting palpebral fissures	+	−	+	+	−
Strabismus	+	+	+	−	−
Esotropia	+	−	−	−	−
Round face	+	+	+	+	+
Philtrum abnormalities	+	+	+	−	+
Coarse hair	−	+	+	−	+
Short neck	+	−	+	+	+
Abnormal ears	+	+	+	−	+
Nasal abnormalities	+	+	+	+	+
Anteverted nares	+	−	+	−	+
Palate (arched or cleft)	−	+	−	−	+
Micrognathia	+	−	+	−	+
Midface hypoplasia	−	−	−	+	−
Hypertelorism	−	+	−	−	−
Hearing deficit	−	−	−	+	−
Thin upper lip	−	−	−	−	+
Digital anomalies	+	+	+	−	+
Macrocephaly	−	−	+	+	−
Dolichocephaly	+	−	−	−	−
Plagiocephaly	−	+	−	−	+
Intellectual disability	+	+	+	+	+
Attention deficit hyperactivity disorder	+	−	−	−	−
Sensory processing disorder	+	−	−	−	−
Speech delay	+	+	−	+	+
Poor motor coordination	+	+	+	+	+
Developmental delay	+	+	+	+	+
Other behavioural issues	+	+	+	+	+
Vertebral anomalies	−	+	−	−	+
Renal anomalies	−	+	−	+	+
Cardiac malformations	+	+	−	−	+
Genital anomalies	−	+	−	−	+
Skeletal anomalies	+	+	−	−	+
Dental abnormalities	+	−	−	−	+

^*∗*^Grammatico et al. is a case series involving two patients. Adapted from Grammatico et al. [8], Bartolini et al. [9], Trachoo et al. [12], and Chaabouni et al. [13].
